# The early development of neutron diffraction: science in the wings of the Manhattan Project

**DOI:** 10.1107/S0108767312036021

**Published:** 2012-12-05

**Authors:** T. E. Mason, T. J. Gawne, S. E. Nagler, M. B. Nestor, J. M. Carpenter

**Affiliations:** a Oak Ridge National Laboratory, Oak Ridge, TN 37831 USA; b Argonne National Laboratory, Argonne, IL 60439, USA

**Keywords:** neutron diffraction, Manhattan Project

## Abstract

Although neutron diffraction was first observed using radioactive decay sources shortly after the discovery of the neutron, it was only with the availability of higher intensity neutron beams from the first nuclear reactors, constructed as part of the Manhattan Project, that systematic investigation of Bragg scattering became possible. Remarkably, at a time when the war effort was singularly focused on the development of the atomic bomb, groups working at Oak Ridge and Chicago carried out key measurements and recognized the future utility of neutron diffraction quite independent of its contributions to the measurement of nuclear cross sections. Ernest O. Wollan, Lyle B. Borst and Walter H. Zinn were all able to observe neutron diffraction in 1944 using the X-10 graphite reactor and the CP-3 heavy water reactor. Subsequent work by Wollan and Clifford G. Shull, who joined Wollan’s group at Oak Ridge in 1946, laid the foundations for widespread application of neutron diffraction as an important research tool.

## Abstract 2

Early neutron diffraction experiments performed in 1944 using the first nuclear reactors are described.

## 1. Introduction

Neutron diffraction is now a widely used and important technique for determining the structure of materials. This contribution to the special issue celebrating the centenary of Bragg’s law examines the crucial early experiments that paved the way for this use of neutrons. Since these experiments were carried out under the auspices of the Manhattan Project, the publication of their results was delayed until after the end of World War II, and the open literature does not fully reflect the excitement and challenge of the early work. The prerequisite for these experiments was the existence of sufficiently intense sources of neutron beams. The following discussion covers the development of the first sources, the key experiments carried out at Manhattan Project facilities in Tennessee and Illinois, and the subsequent early evolution of the field.

The diffraction of neutrons was first demonstrated using radioisotope-driven (α, *n*) sources in experiments carried out by von Halban & Preiswerk (1936 ▶) and Mitchell & Powers (1936 ▶) soon after James Chadwick’s discovery of the neutron in 1932 (Chadwick, 1932 ▶), but the low intensity of early neutron sources prevented further exploitation of the effect. These first diffraction experiments were aimed at demonstrating properties of neutrons themselves, namely the wave–particle duality of the neutron.

Chadwick’s discovery, in combination with the experiments of Frédérick and Irène Joliot-Curie (Joliot & Curie, 1934 ▶), also inspired Enrico Fermi’s systematic investigation of ‘the effects of the bombardment with neutrons’ (Fermi, 1938 ▶), which led to a series of papers on neutron activation (summarized in Fermi, Amaldi, D’Agostino *et al.*, 1934 ▶) and to the discovery that the presence of hydrogenous materials around the primary neutron source greatly enhances the radioactivity produced by neutrons. From these experiments, Fermi inferred that neutrons were being moderated, or slowed down, in multiple collisions with protons, the effect known today as neutron thermalization. He also coined the term ‘slow neutron’, which is applicable to neutrons with wavelengths comparable with interatomic spacing and hence appropriate for diffraction (Fermi, Amaldi, Pontecorvo *et al.*, 1934 ▶).

## 2. Development of chain-reacting piles

Fermi emigrated to the United States in 1939, arriving just as the news of Hahn and Strassmann’s discovery of nuclear fission began to spread. Fermi took part in early experiments on the fission of uranium by neutron bombardment at Columbia University (Anderson *et al.*, 1939 ▶) and pursued the development of subcritical ‘exponential piles’ of uranium oxide powder in a graphite matrix (Weinberg, 1994 ▶, p. 15). During the summer of 1939, he and Leo Szilard exchanged correspondence on the use of graphite as a neutron moderator for a nuclear chain reaction (Anderson, 1965 ▶). Both Fermi and Szilard made approaches to the United States government regarding the potential military importance of this work (Smyth, 1945 ▶, pp. 46–47), and in October 1939 President Franklin Roosevelt appointed an Advisory Committee on Uranium.

The Uranium Committee would soon evolve into the Manhattan Project, the World War II effort to develop atomic weapons. In mid-January 1942 Arthur H. Compton, one of the leaders of the project, directed the consolidation of work on the chain reaction at the University of Chicago (Smyth, 1945 ▶, p. 79), and in April 1942 the Columbia University group led by Fermi moved to the Metallurgical Laboratory at the University of Chicago.

Fermi’s status as a member of the Manhattan Project was complicated by his Italian citizenship. From 8 December 1941 until 12 October 1942 he was regarded by the United States military as an ‘enemy alien’, and his movements and access to information were restricted (Segrè, 1972 ▶; Wellerstein, 2012 ▶). Harold Agnew has speculated that this situation contributed to the listing of Fermi as only a consultant on the Metallurgical Laboratory’s organization chart (Agnew, 2012 ▶) despite his widely acknowledged theoretical, experimental and practical contributions to all aspects of the project.

Construction of the first nuclear reactor, CP-1 (Chicago Pile 1), began under Fermi’s direction in November 1942. The successful operation of CP-1 in December 1942 was a key step in the Manhattan Project, but it also marked the beginning of a new era in neutron scattering. The moderation of neutrons, first with high-purity graphite and later with heavy water, was essential to sustaining a nuclear chain reaction, but it also meant that the slow neutrons needed for diffraction studies would be available in large enough numbers to enable the detailed studies described in this paper.

The Metallurgical Laboratory at Chicago was the first of the three major sites of the Manhattan Project’s plutonium program. The second was Clinton Laboratories, the plutonium semiworks (pilot plant) at Site X in Tennessee, and the third was the Hanford Engineer Works in Washington state, code named Site W, where the plutonium production facilities were constructed. Three major laboratories evolved from these sites: Argonne National Laboratory (ANL) from the ‘Met Lab’ at Chicago, Oak Ridge National Laboratory (ORNL) from Clinton Laboratories, and Pacific Northwest National Laboratory (PNNL) from Hanford.

The graphite-moderated X-10 pile (later known as the Oak Ridge Graphite Reactor) began operations at Clinton Laboratories under Fermi’s supervision on 4 November 1943, and in May 1944 a heavy-water reactor, CP-3, was started up at the original site of ANL, the Argonne Woods area of the Palos Forest Preserve south of Chicago, where the CP-1 reactor had been reassembled as CP-2. As Clifford G. Shull noted in a talk presented in 1976: ‘Both of these neutron sources were used through the war-time period for obtaining critically needed physical and technical data of interest to the Manhattan Project. Among these activities was the use of single-axis spectrometers and Fermi-type choppers for selecting monoenergetic beams of slow neutrons for cross-section work’ (Shull, 1976 ▶).

Yet even during the Manhattan Project, and particularly once plutonium production was under way at Hanford, these neutron sources were also exploited for more fundamental explorations of the interactions of neutrons with matter.

## 3. Neutron diffraction experiments at Clinton

**Figure 1:**
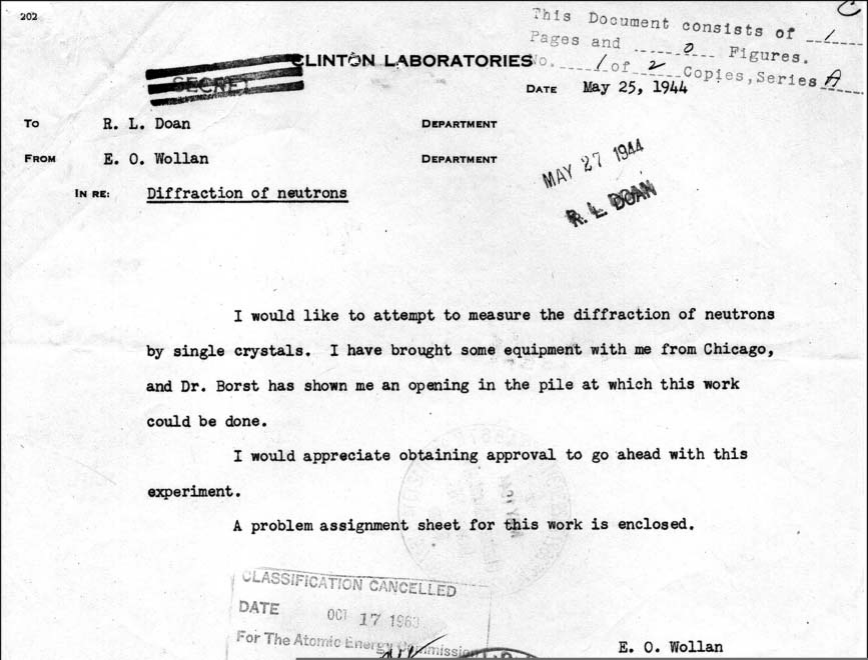
Memo from Wollan to Richard L. Doan requesting approval for neutron diffraction measurements at the X-10 pile.

In May 1944 Ernest O. Wollan sent a memorandum (reproduced in Fig. 1 ▶) to R. L. Doan, associate director for research at Clinton Laboratories, requesting approval for an attempt to measure the diffraction of neutrons by single crystals at the X-10 pile (Wollan, 1944 ▶).

Wollan had completed his PhD under Compton at the University of Chicago in 1929. His thesis work involved X-ray scattering, including measurements utilizing Bragg diffraction from calcite (Wollan, 1928 ▶) and MgO (Wollan, 1930 ▶). At the request of Compton and Fermi, he joined the Metallurgical Project at Chicago in January 1942 and was present at the start-up of both CP-1 in December 1942 and the X-10 pile in November 1943 (Wollan, 1980 ▶).

**Figure 2:**
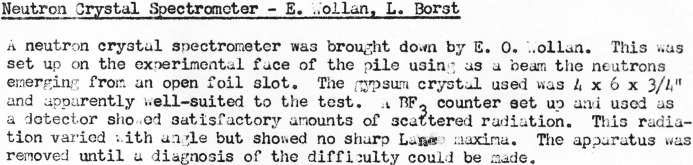
Report of installation of Wollan’s neutron crystal spectrometer and unsuccessful attempts to observe neutron diffraction in May 1944.

As indicated in Wollan’s May 1944 memorandum, he had previously consulted with Lyle B. Borst about the availability of experimental openings in the pile. Borst, who had joined the Metallurgical Project in 1941 after completing his PhD in chemistry at Chicago, was by this time coordinator for experiments in Building 105 (which housed the X-10 pile) at Clinton Laboratories. Neutron diffraction was added to the list of new problems assigned to Clinton in May 1944 (Smyth, 1945 ▶, p. 145), and Wollan’s instrument was installed, but early attempts were unsuccessful, as indicated in Fig. 2 ▶ (Wollan & Borst, 1944 ▶).

**Figure 3:**
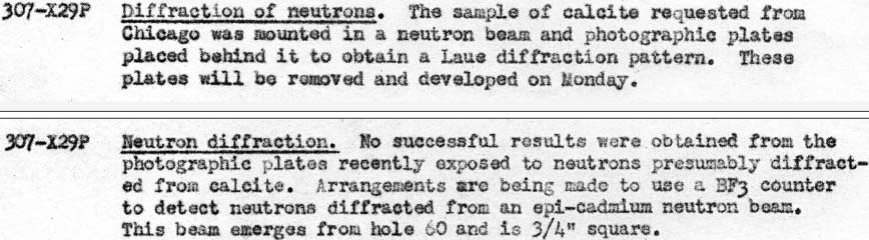
July 1944 reports by Borst (top: 8 July; bottom: 12 July) on attempts to obtain a Laue diffraction pattern.

During the summer both Borst and Wollan continued their attack on this problem. In the daily reports for 8 July and 12 July (Fig. 3 ▶), Borst recorded additional unsuccessful attempts (Borst, 1944 ▶). Later that month, Borst’s colleague A. J. Ulrich reported ‘indications of a reflected beam’ from a calcite crystal ‘set up in an appropriate spectrometer’ (Ulrich, 1944 ▶). In a mid-September report, Ulrich and Borst stated that ‘the spectral distribution of neutrons emerging from the center of the Clinton pile has been determined using a calcite crystal spectrometer and BF_3_ counter as detector’, but they also mentioned ‘the poor resolving power of the present instrument’ as an impediment to accurate determination of angles, as well as their hopes for an improvement in resolving power from a ‘double circle’ (*i.e.* two axis) spectrometer recently supplied by Chicago (Ulrich & Borst, 1944 ▶).

**Figure 4:**
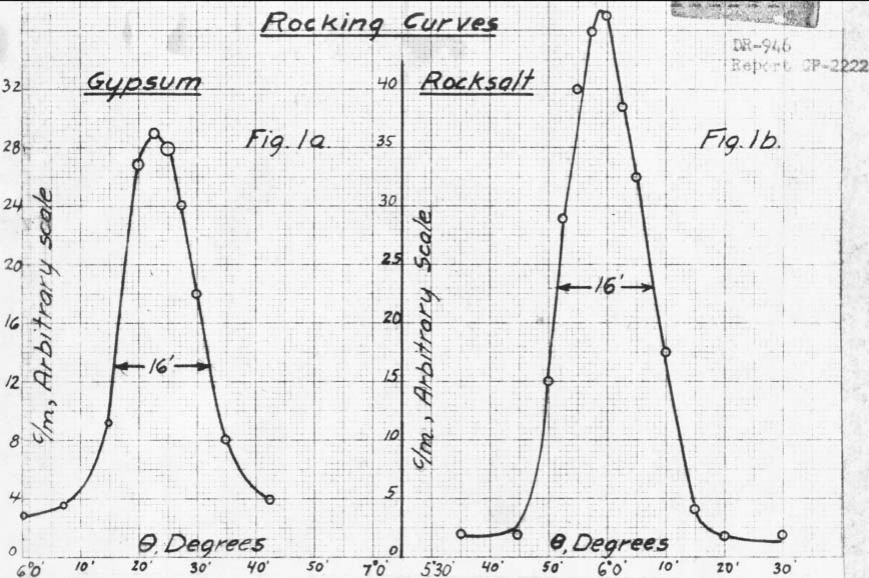
Hand-plotted rocking curves for Bragg scattering from single crystals at the X-10 pile, obtained by Wollan & Borst in December 1944 with improved equipment installed on 2 December.

Wollan’s efforts were likely hampered first by the transfer of his group (General Physics Section II) from Chicago to Clinton in August 1944 and then by his engagement in resolving the xenon poisoning problem at Hanford (Moak, 1976 ▶). By December 1944, however, Wollan and Borst were able to report success, providing to the Physics Division monthly report the two rocking curves, one for a gypsum crystal and one for an NaCl crystal, shown in Fig. 4 ▶ (Wollan & Borst, 1945 ▶).

## 4. Neutron diffraction experiments at Argonne

The CP-3 reactor, designed by Eugene Wigner and built under the direction of Walter H. Zinn, was initially envisioned as a backup for the Hanford reactors (Weinberg, 1994 ▶, pp. 31–33; Holl *et al.*, 1997 ▶, p. 28). Since it used heavy water (code named P-9) as a moderator, it was also called the P-9 machine. By July 1944, CP-3 was operating under full power, and Fermi and Zinn were working to optimize the collimation of the neutron beam from its thermal column and measure the index of refraction for thermal neutrons (Fermi & Zinn, 1944 ▶). The Smyth report would subsequently note that “The very high intensity beam of neutrons produced by this pile has been found well-suited to the study of ‘neutron optics’, *e.g.*, reflection and refraction of neutron beams” (Smyth, 1945 ▶, p. 140).

Zinn, a Canadian who received his PhD in nuclear physics from Columbia University in 1934, had carried out experiments at Columbia with Szilard on the emission of neutrons from the fission of uranium (Szilard & Zinn, 1939 ▶) before transferring to Chicago in 1942 with the other members of the Columbia nuclear physics group. He played a major role in the design and construction of CP-1 and subsequently became the first director of ANL.

**Figure 5:**
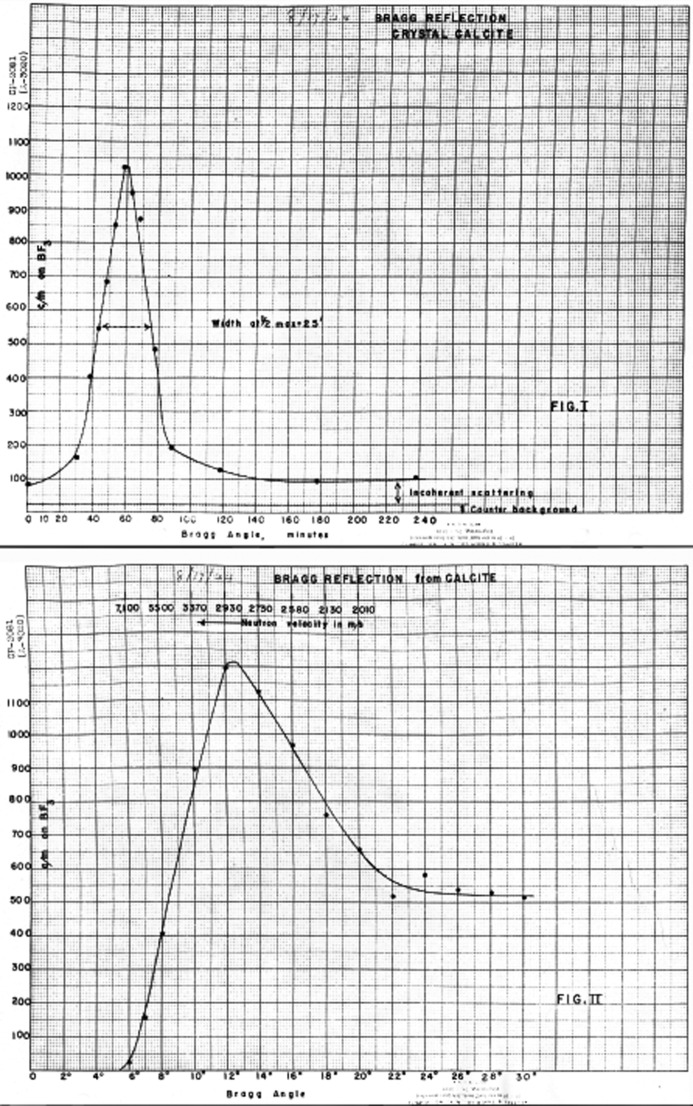
Bragg reflection of neutrons on CP-3, obtained in August 1944. Top: typical crystal rocking curve; bottom: results of an exploration of the thermal neutron spectrum, with a background of 150 counts per minute subtracted.

**Figure 6:**
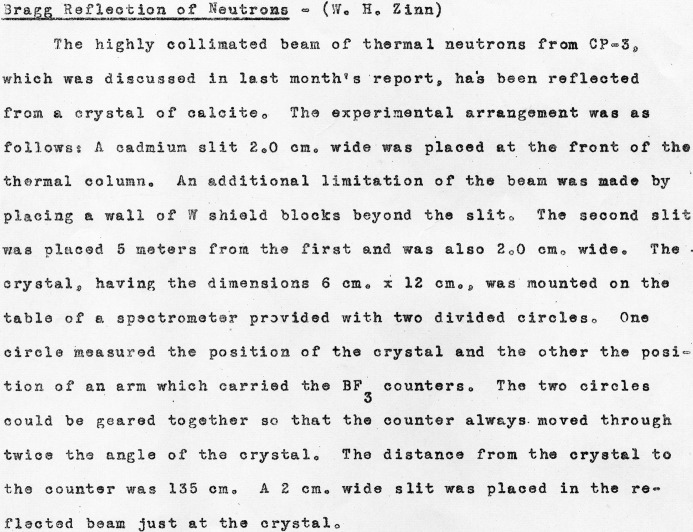
Zinn’s description of the experimental arrangement used to reflect a highly collimated beam of thermal neutrons from a calcite crystal.

By the end of August 1944, Zinn was able to report that ‘The highly collimated beam of neutrons from CP-3, which was discussed in last month’s report, has been reflected from a crystal of calcite’ and to provide both ‘a typical crystal rocking curve’ and a diffraction measurement illustrating the spectral distribution of neutrons, shown in Fig. 5 ▶ (Zinn, 1944 ▶). His description of the experimental arrangement is reproduced in Fig. 6 ▶.

**Figure 7:**
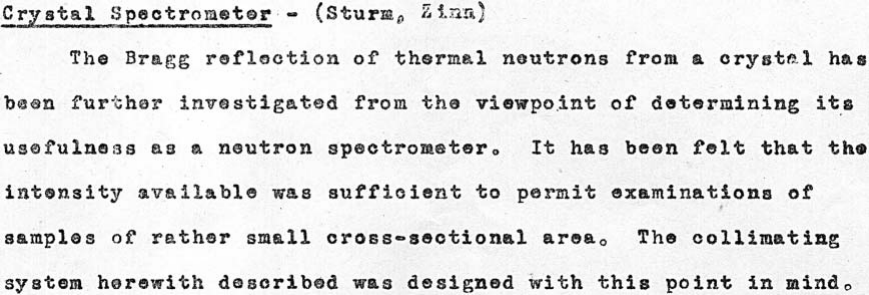
Report of continuing work on CP-3 by Sturm & Zinn in November 1944 to determine the usefulness of the Bragg reflection of thermal neutrons from a crystal as a neutron spectrometer.

Following Fermi’s departure for Los Alamos in September 1944, Zinn continued these investigations, working with William Sturm. In November 1944 they reported on further investigations of the Bragg reflection of thermal neutrons from a crystal (see Figs. 7 ▶ and 8 ▶) ‘from the viewpoint of determining its usefulness as a neutron spectrometer’ (Sturm & Zinn, 1944 ▶). The positive nature of their findings may be inferred from the 1946 photograph of Zinn with the crystal spectrometer at CP-3 shown in Fig. 9 ▶.

In a 1981 interview, Frederick Seitz recalled that ‘Fermi did a few rather basic experiments on diffraction…. Then Zinn picked this up and I worked with him on the theoretical side’, collaborating with M. L. Goldberger (Seitz, 1981 ▶). In mid-November 1944 Goldberger and Seitz reported on their examination of ‘the equations for the elastic scattering of neutrons by a single crystal (*i.e.* Laue–Bragg scattering)’ (Goldberger & Seitz, 1944 ▶). This early report attributed ‘the recent experiments on the scattering of neutrons by single crystals’ only to Zinn and Borst. In a 1947 open literature publication based on this report (Goldberger & Seitz, 1947 ▶), the attribution was expanded to include Fermi at Argonne and Wollan at Clinton, and a footnote indicates that ‘some of the results derived in this report, particularly those for the index of refraction, were derived earlier by Fermi’. The footnote also mentions that ‘Fermi’s measurements of the total reflection of thermal neutrons by graphite and subsequent measurements of Bragg scattering by Zinn and Borst furnished the incentive for much of the work described here’, so perhaps Seitz’s memory, some four decades after the fact, was not entirely reliable.

## 5. Communication and publication of results

As noted by Moak (1976 ▶), there was little compartmentalization of information within the scientific groups of the Metallurgical Project, but all of the early Clinton and Argonne reports on neutron diffraction experiments were classified. Comprehensive guidelines for declassification of Manhattan Project information were developed soon after the end of World War II, but the release of information proceeded slowly, as documents were declassified individually rather than on the basis of their subject matter (Quist, 2002 ▶). The resulting delays create some confusion about the priority of results.

In the minutes of the June 1946 meeting of the American Physical Society (APS) in Chicago, Karl K. Darrow, APS secretary, noted that “This will be long remembered as the great nuclear-physics meeting at which a large amount of the work done under the famed ‘Manhattan Project’ was finally brought to light” (Darrow, 1946 ▶). Fermi’s name appears twice on the list of invited papers for this meeting: as the sole author of a paper on elementary pile theory, and as one of a host of contributors to ‘a critical survey of neutron cross sections’ presented by H. H. Goldsmith (Darrow, 1946 ▶, p. 100). Fermi is also listed as a coauthor of three contributed papers: one on ‘production of low energy neutrons by filtering through graphite’, with Herbert L. Anderson and Leona Marshall (Anderson *et al.*, 1946*a* ▶); one on reflection of neutrons on mirrors, with Zinn (Fermi & Zinn, 1946 ▶); and one on determining the ‘phase of neutron scattering’ for various elements, with Marshall (Fermi & Marshall, 1946 ▶). Zinn also contributed a paper on the Bragg reflection of neutrons by a single crystal (Zinn, 1946 ▶). Borst contributed a paper on diffraction of neutrons and neutron absorption spectra (Borst *et al.*, 1946*a* ▶). Much of this work was subsequently published in the open literature (see, *e.g.*, Anderson *et al.*, 1946*b* ▶; Borst *et al.*, 1946*b* ▶; Fermi & Marshall, 1947 ▶; Zinn, 1947 ▶), often with notes indicating that it had been accomplished during the Manhattan Project.

Wollan was apparently unable to obtain clearance for his work on neutron diffraction in time for the June 1946 APS meeting. His name appears only as coauthor of a contributed paper describing a study of ‘delayed neutron energies from U^235^ fission products’ (Burgy *et al.*, 1946 ▶) and as a contributor to the Goldsmith paper. At the September 1946 APS meeting in New York, however, Wollan and his colleagues presented an invited paper on their application of a bent crystal neutron spectrometer to measurements of resonance absorption (Sawyer *et al.*, 1946 ▶). This instrument is shown in Fig. 10 ▶.

Also missing from the literature in 1946 were the results of investigations of neutron diffraction from liquids and polycrystalline samples using this instrument (Sawyer & Wollan, 1946 ▶). Shull was shown the first neutron diffraction patterns of polycrystalline NaCl and light and heavy water when he visited Oak Ridge in the spring of 1946 (Shull, 1995 ▶), but this work was not published until 1948 (Wollan & Shull, 1948 ▶; Shull *et al.*, 1948 ▶), and the internal report in which it was documented was not declassified until 1956.

**Figure 8:**
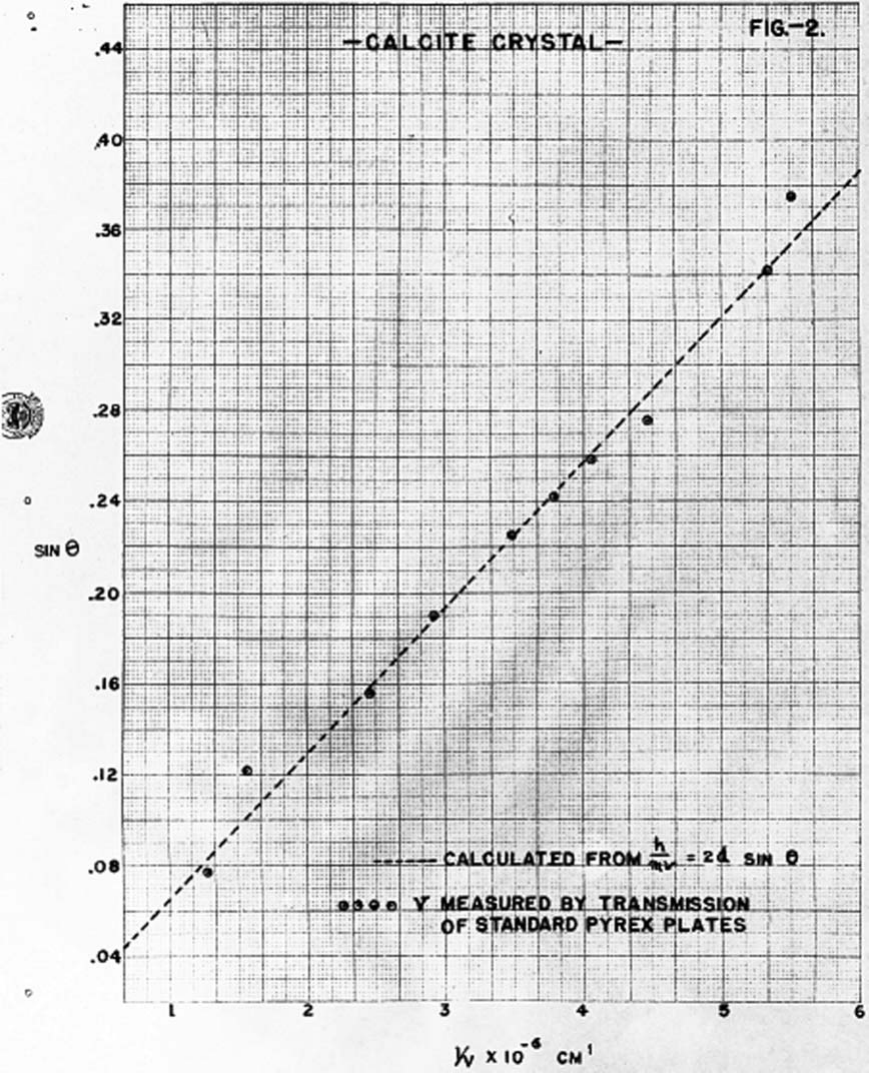
Results of measurements made by Sturm & Zinn in November 1944. The effective velocity of the neutrons was determined by measuring transmission at various angles through a standardized Pyrex plate. The reciprocal of the measured velocity is plotted (points) against the sine of the crystal angle. Also shown (dashed line) is the reciprocal of the velocity as obtained from the relationship 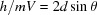.

**Figure 9:**
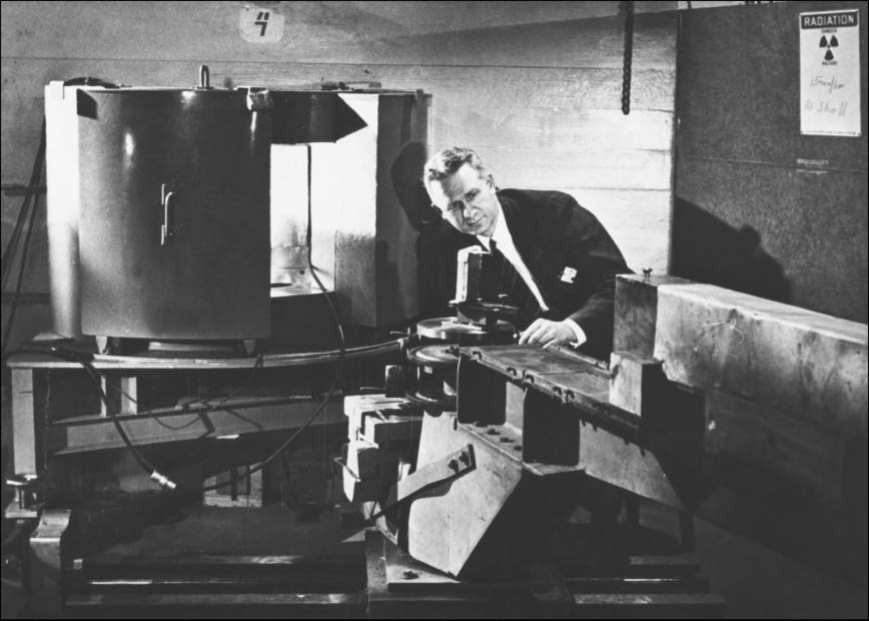
Walter Zinn at the crystal spectrometer at the CP-3 reactor at ANL in 1946. These were the days when scientists wore suits and ties to work (Courtesy ANL Graphic Arts Archives).

**Figure 10:**
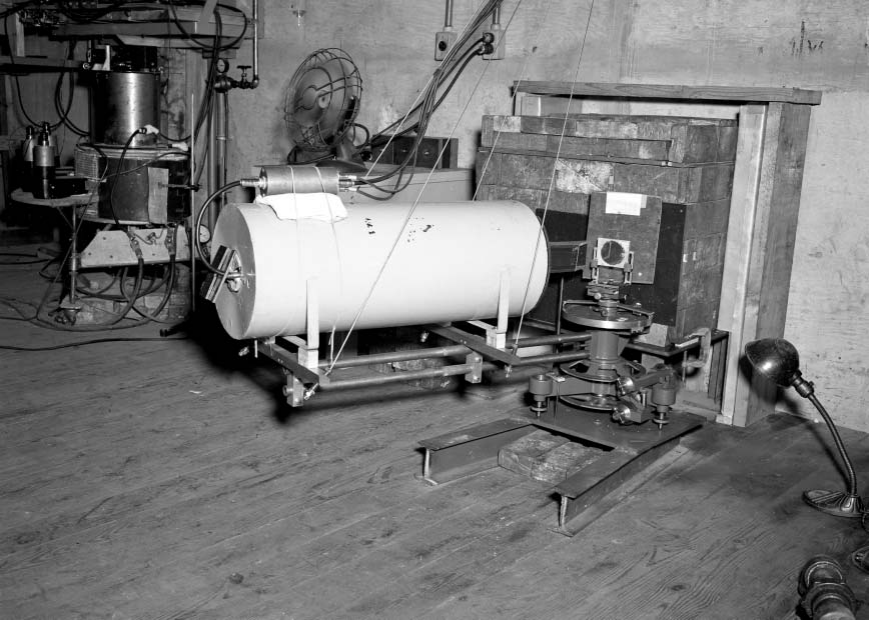
Wollan’s crystal spectrometer at the graphite reactor in Oak Ridge in 1946.

The published literature following the war and the subsequently declassified experimental reports and log entries in Figs. 1–8 ▶ ▶ ▶ ▶ ▶ ▶ ▶ ▶ establish that between August and December 1944 the parallel efforts of Borst, Wollan and Zinn demonstrated the key features of Bragg scattering: the variation with angle of incidence to a crystal plane of the scattered intensity (the rocking curves) and the linear relationship of inverse velocity (and hence wavelength) with the sine of the scattering angle (Bragg’s law). This explicit confirmation of diffraction, which had only been inferred from the earlier measurements with radioactive sources, was enabled by the dramatic increase in neutron flux realised with the first chain-reacting piles.

## 6. Outcomes

**Figure 11:**
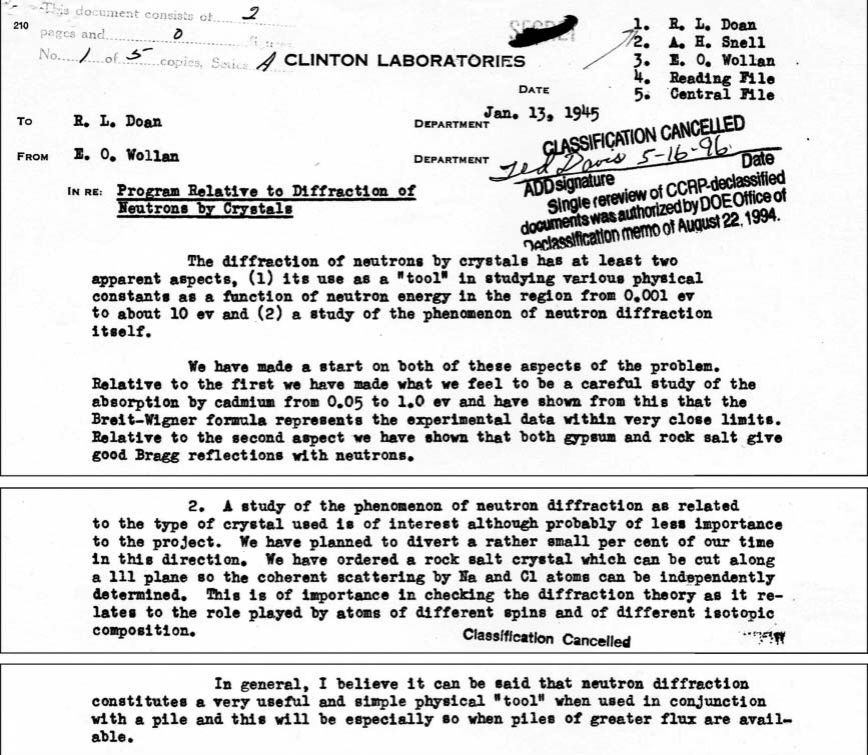
Excerpts from Wollan’s memo to Doan summarizing the results of measurements carried out at the graphite reactor in late 1944.

Wollan appears to have been the first to recognize the possible utility of neutron diffraction as a phenomenon of interest in its own right, as opposed to a means of producing monochromatic beams for use in cross-section measurements (see Fig. 11 ▶). Certainly he was the only one of the research leaders discussed here who continued to focus on exploring this phenomenon. By mid-1946, Borst was on his way to the new Brookhaven National Laboratory to help design its first reactor (Westwick, 2003 ▶). Zinn, who had become acting director of the Argonne Branch of the Metallurgical Laboratory in 1944, served as director of ANL from 1946 to 1956, leading the design and construction of a series of reactors. Fermi returned from Los Alamos to Chicago in 1946, where he pursued a broad program of experimental and theoretical work in nuclear and elementary particle physics until his untimely death in 1954.

**Figure 12:**
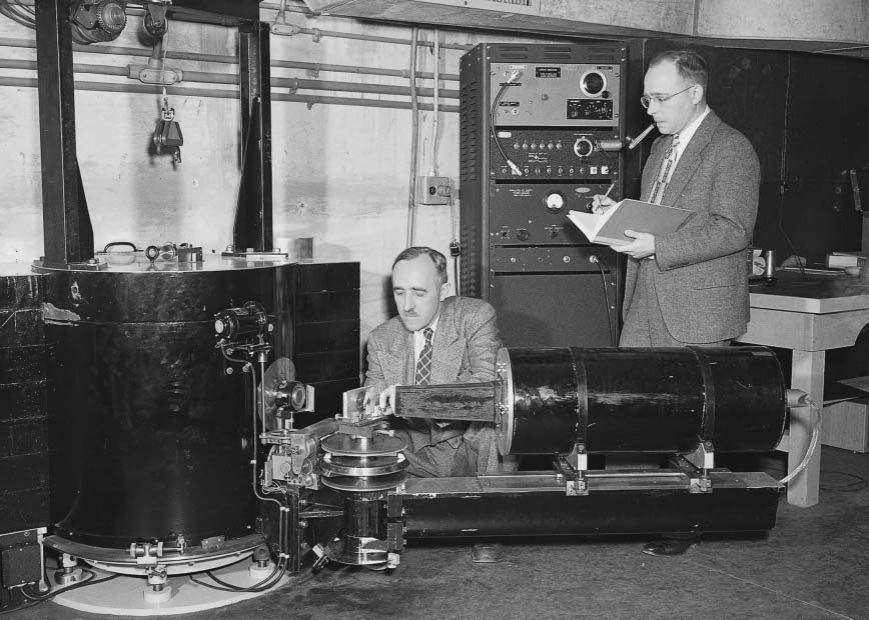
Wollan and Shull taking data on a double-crystal neutron spectrometer, installed on the south face of the ORNL graphite reactor in 1949.

Wollan remained at Oak Ridge, where with Shull, who joined him in 1946, he began a systematic investigation of the fundamentals of the diffraction of thermal neutrons by crystalline powders. Wollan and Shull are shown in Fig. 12 ▶ with a double-crystal neutron spectrometer built to Wollan’s specifications in 1949. This instrument was, according to Shull, ‘the first prototype of present-day neutron spectrometers, having components that were built for specific neutron use rather than being improvised from x-ray units’ (Shull, 1995 ▶). In April 1948 they reported the results of diffraction measurements on diamond, graphite, Al, Na, NaBr, NaCl and NaF, as well as measurements made ‘on a number of other crystals with the purpose of determining the phase of scattering’, extending the earlier work of Fermi & Marshall (Wollan & Shull, 1948 ▶). With G. A. Morton and W. L. Davidson, they also reported the results of studies on the diffraction of neutrons in NaH and NaD, which they identified as ‘a promising method of obtaining information on the location of hydrogen positions in crystalline and molecular structures’ (Shull *et al.*, 1948 ▶).

In June 1948 Wollan and Shull published an overview in *Science* that compared X-ray, electron and neutron diffraction (Shull & Wollan, 1948 ▶). They concluded, somewhat diffidently, that ‘The newly developing field of neutron diffraction would appear to have advantages over the older fields of X-ray and electron diffraction in some problems involved in the determination of crystal and molecular structures’, and they were careful to point out that ‘the very limited facilities for work in this field which result from the requirement of a chain-reacting pile as a source will make progress less rapid than in the corresponding fields of X-ray and electron diffraction, for which adequate sources can be procured by any laboratory’.

By 1949, however, Alvin Weinberg was able to state that neutron diffraction ‘has become an extraordinarily powerful tool for the investigation of the structure of hydrogen-containing crystals’. He predicted that ‘the use of neutron diffraction for the systematic study of crystals and molecular structure is apt to remain a most important basic scientific function of the national laboratories’ (Weinberg, 1949 ▶). Although this prediction was based in part on the high cost of nuclear reactors, it was accurate in that neutron diffraction soon became a valuable scientific technique for determining the structures and lattice spacings of materials. The use of single-crystal neutron diffraction data for crystal structure determination was demonstrated in 1951 (Peterson & Levy, 1951 ▶), and within a decade neutron diffraction was also being used for studies in nuclear physics, crystal dynamics and investigations of magnetism at the atomic level (Wilkinson *et al.*, 1961 ▶).

In retrospect, neither Wollan and Shull nor Weinberg could have anticipated the profound and far-reaching impact of neutron diffraction studies. Measurements first with powder samples (Wollan *et al.*, 1949 ▶) and subsequently with single crystals (Peterson & Levy, 1957 ▶) established that the crystal structure of ice obeyed the rules proposed earlier by Linus Pauling (Pauling, 1935 ▶) and definitively ruled out several competing theories. In the early 1960s, the United Kingdom Antarctic Place-names Committee assigned the names of researchers who determined the structure of ice crystals to several features in Antarctica’s Crystal Sound, including the Bragg Islands, the Pauling Islands, Levy Island, Wollan Island and Shull Rocks (Cruickshank, 2000 ▶). (These features may be located using the USGS Geographic Names Information System, http://geonames.usgs.gov/antarctic/index.html.) Condensed matter theorist and Nobel laureate Phillip Anderson has recalled (Anderson, 2011 ▶) how the first neutron diffraction measurement of antiferromagnetic Bragg peaks (Shull *et al.*, 1951 ▶) came as a complete surprise, and the discovery was instrumental in leading to the concept of ‘broken symmetry’ that is important in cosmology and particle physics as well as condensed matter physics.

These and other important contributions were ultimately recognized in 1994, when the Nobel Prize in Physics was awarded to Shull and Bertram N. Brockhouse, who pioneered inelastic neutron scattering at Chalk River Laboratories in Canada in the 1950s and 1960s, ‘for pioneering contributions to the development of neutron scattering techniques for studies of condensed matter’. Shull, who was recognized specifically ‘for the development of the neutron diffraction technique’, concluded his Nobel lecture with a tribute to ‘the association, collaboration, and close friendship’ that he enjoyed with Wollan, who died in 1984, and expressed his deep regret that Wollan had not lived long enough to share in the honors that had come to him (Shull, 1995 ▶). Shull further honored his colleague by inviting members of Wollan’s family to attend the Nobel Prize ceremony in Stockholm as his guests.

In the years following the pioneering work reported here, advances in the use of neutrons as a probe of materials have followed not only from increases in reactor power, but also, and more importantly, from the refinement of instruments and techniques. The addition of accelerator-based neutron sources to the nuclear reactors developed during the Manhattan Project has taken neutron diffraction to a new level, thus verifying Wollan’s early view of this technique as ‘a very useful and simple physical tool’.
